# Microbiota responses to environmental stress in mobile and sessile marine invertebrates: evidence for the effect of dissolved oxygen variations

**DOI:** 10.3389/fmicb.2026.1764313

**Published:** 2026-05-13

**Authors:** Valentina Valenzuela-Muñoz, Fabián J. Tapia, Cristian Gallardo-Escárate, Mireia Mestre, Sergio A. Navarrete, Marcelo H. Gutiérrez, María F. Morales-Rivera, Gerdhard L. Jessen

**Affiliations:** 1 Center for Oceanographic Research COPAS Coastal, Universidad de Concepción, Concepción, Chile; 2 Departamento de Oceanografía, Facultad de Ciencias Naturales y Oceanográficas, Universidad de Concepción, Concepción, Chile; 3 Museo Nacional de Ciencias Naturales (MNCN-CSIC), Madrid, Spain; 4 Estación Costera de Investigaciones Marinas, Pontificia Universidad Católica de Chile, Las Cruces, Chile; 5 Instituto de Ciencias Marinas y Limnológicas, Universidad Austral de Chile, Valdivia, Chile

**Keywords:** 16S rRNA, adaptation, crustacea, dissolved oxygen, microbiota, Pacific Ocean, tunicate

## Abstract

Microbiomes can modulate plant and animal responses to environmental fluctuations, revealing their central role in global biogeochemical cycles and the physiology of individual organisms, and in how these fluctuations are modulated. In marine ecosystems, Eastern Boundary Upwelling Systems (EBUS) allow us to examine host-microbiome dynamic interactions that respond to environmental variation, offering critical insights into the potential impacts of global environmental change. This study investigates the microbiota response of two marine invertebrates that co-occur at sites with strong synoptic and seasonal fluctuations in temperature and dissolved oxygen concentration between winter and summer. Specimens of the sessile marine tunicate *Pyura chilensis* (“Piure”) and the porcelain crab *Allopetrolisthes punctatus* (“Changai”) were collected at about 10 m depth under contrasting oxygen regimes in summer and winter at Chome, central Chile. Microbiota from gills and digestive glands were assessed via 16S rRNA Nanopore sequencing, which showed clear differences in bacterial community structure and composition between species and across summer and winter surveys. Functional predictions suggest that shifts in the microbiota are primarily associated with variations in exposure to hypoxic waters induced by intensified upwelling. This suggests that the microbiomes may positively influence host performance under environmental stress, enhancing resistance to low dissolved oxygen conditions.

## Introduction

Thanks to advances in molecular and data analysis techniques, the last decade has witnessed a revolution in our understanding of the critical role microbial communities play in the biogeochemical cycles, on which all life depends (Hallin and Bodelier, 2020; Knight et al., 2018), and in the physiology and homeostasis of individual organisms. The discovery that microbial communities inhabiting other organisms (“internal microbiota”) can modulate plant and animal responses to environmental fluctuations, once thought to be largely independent of microbes, is reshaping our conception of what constitutes an individual, how species’ niches should be defined, and the units upon which natural selection operates (Dai et al., 2022; Johnson and Marín, 2025). We are only beginning to understand how natural environmental variability influences interactions among microbial communities and their hosts. Understanding how microbiotas respond to the environment is essential for anticipating the potential effects of anthropogenic and climatic disturbances. In marine ecosystems, key environmental variables such as seawater temperature, pH, and dissolved oxygen are being altered by climate change and other global drivers. In this context, Eastern Boundary Upwelling Systems (EBUS), where the wind-driven onshore transport of subsurface waters produces large natural fluctuations in these biologically critical variables, offer an exceptional opportunity to assess the impacts of environmental variability on microbiota communities. Indeed, besides temperature and nutrient levels, large fluctuations in pH and dissolved oxygen concentration characterize upwelling shores (Chan et al., 2017; De La Maza and Farías, 2023; Muñoz et al., 2025) and provide clues to global phenomena such as ocean acidification and deoxygenation (Breitburg et al., 2018; Doney et al., 2020; Fusi et al., 2025).

The coastal ocean is a heterogeneous and temporally variable environment, with complex circulation patterns and oceanographic conditions, strongly influenced by coastal bathymetry and the interaction with wind forcing and riverine inputs (Kirincich et al., 2005; Sobarzo et al., 2016). Physical variables such as temperature, salinity, dissolved oxygen (DO), and pH fluctuate widely over temporal scales that ranging from seasons to days, hours, and even minutes (Adams et al., 2013; Chan et al., 2017; Muñoz et al., 2025; Tapia et al., 2014; Walter et al., 2014). Over the past decade, extreme environmental variability in the coastal ocean has been increasingly reported, including in upwelling nearshore ecosystems (Grantham et al., 2004; Hernández-Miranda et al., 2012). While temperature has remained relatively stable along these shores (Aguirre et al., 2018; García-Reyes et al., 2022; Sydeman et al., 2014), several environmental changes associated with climate change have been reported, including shifts in primary productivity and in the intensity and frequency of hypoxic events (Barth et al., 2024; De La Maza and Farías, 2023; Fusi et al., 2025). The ecosystem response to hypoxia depends on the timing, predictability, and intensity of oxygen depletion. Sustained hypoxia in nearshore areas is generally accompanied by a loss of biodiversity and ecosystem functions, and by increased mortality of benthic organisms, followed by some degree of renewed colonization upon return to normoxia (Diaz and Rosenberg, 2008; Muñoz et al., 2025).

The response of marine metazoans to oxygen depletion has been studied in terms of behavioral, physiological, and metabolic changes (Levin, 2003). Various taxa exhibit different levels of tolerance to low-oxygen conditions (Connolly et al., 2004; Lagos et al., 2017). Among large metazoans, cnidarians and annelids are generally considered most tolerant to hypoxia (Lee et al., 2023; Vaquer-Sunyer and Duarte, 2008), whereas crustaceans and fish are known to be more sensitive to low-oxygen conditions (Levin et al., 2009; Vaquer-Sunyer and Duarte, 2008). Although an organism’s survival under environmental stress, such as a decline in oxygen availability, has been typically viewed as the result of its own capacity to respond or acclimate, it is now known that its microbiota plays a role in responding to environmental changes (Sun et al., 2019; Ullah Khan et al., 2021). Changes in water quality can reshape microbial communities and may contribute to organism health if there is a significant alteration in their microbiota structure and abundance (Khan et al., 2018; Ullah Khan et al., 2021). Given the influence of the microbiota on the physiology and metabolism of marine organisms, microbes are expected to contribute to their adaptation to a changing ocean environment (Apprill, 2017; Sehnal et al., 2021). For instance, exposure to hypoxia has been shown to alter the microbiota of mussels (Montúfar-Romero et al., 2025; Ullah Khan et al., 2021) and fish (Fan et al., 2020; Wang W. Z. et al., 2021; Zheng et al., 2021). Reporting changes in microbiota richness, and an enrichment of microbiota with functions related to methane metabolism, carbon fixation, and key metabolic pathways has been observed in fish exposed to hypoxia (Fan et al., 2020; Liu et al., 2024; Zheng et al., 2021).

In the mussel, *Mytilus coruscus*, changes in their gut microbiota have been triggered by changes in DO levels (Ullah Khan et al., 2021). A recent study of the blue mussel *Mytilus chilensis* gut exposed to hypoxic conditions revealed significant alterations in microbial composition, with a shift toward facultative anaerobes (Montúfar-Romero et al., 2025). Functional analysis indicated a decline in critical microbial functions associated with nutrient metabolism and immune support, potentially jeopardizing the health and survival of the host (Montúfar-Romero et al., 2025). Highlighting the importance of understanding the role of host-microbiota interactions in increasingly altered marine environments, especially in regions impacted by coastal hypoxia. This study focuses on the microbiota of benthic invertebrates from the upwelling coast of central Chile, which experiences large seasonal and shorter-term changes in the oxygen conditions of near-bottom waters (Muñoz et al., 2025). We selected two species of filter feeders with contrasting behavior: the sessile tunicate *Pyura chilensis*, widely distributed along the Chilean coast (Haye et al., 2021), a conspicuous species considered a bioengineer, a dominant competitor of hard substrates, and a species with economic relevance for small-scale aquaculture activity in Chile (Pérez-Valdés et al., 2017; Manríquez and Castilla, 2005). The other specie evaluated in this study is the porcelanid crab *Allopetrolisthes punctatus*, which is highly mobile, locally abundant, but patchily distributed between northern and south-central Chile (18–40°S) (Santa Cruz and Retamal, 2018). This crab shows gregarious behavior, forming dense aggregations that form multi-layered clusters (Viviani et al., 2010). We characterized the gill and gut microbiotas of both species and compared them with the ambient microbiota’s composition and changes between the upwelling season in summer–characterized by low DO levels–and the non-upwelling period in winter, which has higher DO levels. We hypothesized that, like their hosts, the microbiota of mobile and sessile marine invertebrates varied in response to environmental stress (i.e., DO), thereby influencing host adaptation capacity.

## Materials and methods

### Study area

**FIGURE 1:**
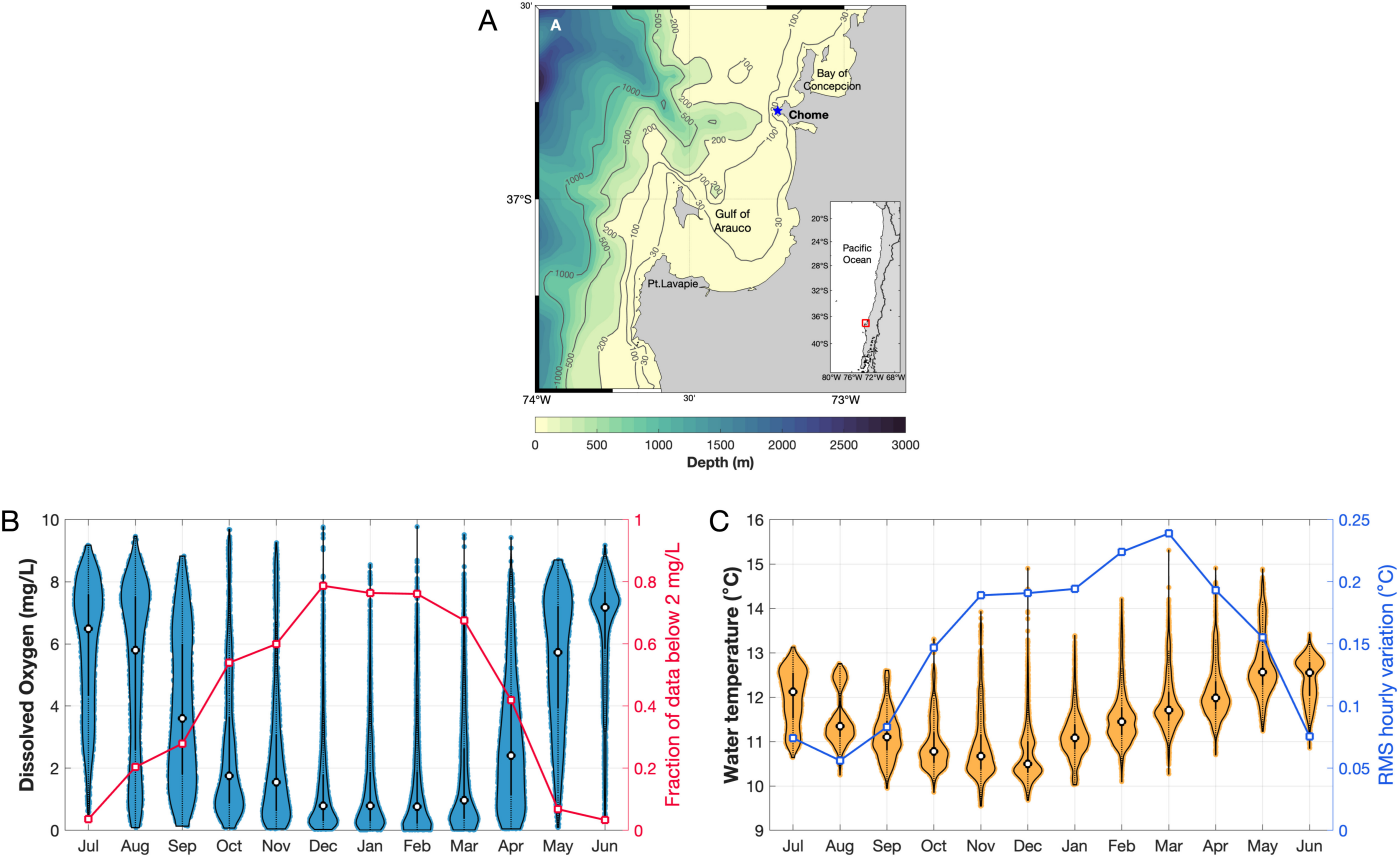
Environmental conditions of the study site. **(A)** Geographic localization of study site – Chome Cove, central Chile (36°46′S, 73°12′W). **(B)** Monthly distributions of dissolved oxygen concentration (left *Y*-axis), line plots show monthly variation in the fraction of data with DO concentrations below 2 mg/L (right *Y*-axis). **(C)** Monthly distributions of temperature in near-bottom waters from data 2024 to 2025, left *Y*-axis showed the temperature in °C, and the right *Y*-axis showed the metric variability in water temperature for hourly (see methods). *X*-axis: are the month from July 2024 to June 2025.

The study was conducted at a nearshore site on the central Chile coast - Chome, 36° 46′ 24′′ S, 73° 12′ 38′′ W (Figure 1A). This small fishing cove is located just north of the Biobío River mouth; its near-bottom conditions are strongly influenced by the seasonality of coastal upwelling and by the interaction between tidal currents and the Biobío submarine canyon (Hernández and Tapia, 2021; Muñoz et al., 2025; Sobarzo et al., 2016). Temporal variability in near-bottom hydrographic conditions, temperature and dissolved oxygen, at Chome was characterized from continuous records gathered from July 2024 to May 2025 using a suite of environmental sensors moored at ca. 2 m above the bottom at a nominal depth of 30 m. The sensor array included loggers for dissolved oxygen and temperature (MiniDOT, PME, USA), pressure (HOBO U20, Onset Computer, USA), and conductivity (HOBO U24, Onset Computer, USA). All loggers were programmed to record data at 10-min intervals and were serviced every 3–4 months. The data gathered from the loggers were quality controlled, curated, and converted into hourly time series prior to the analysis. To visualize the seasonal variability in exposure to hypoxic conditions, the fraction of recorded values below 2 mg/L was computed for each month. The seasonal variability in water temperature, and its co-variation with DO variability, were assessed by computing monthly distributions from all recorded values. Additionally, the RMS (root mean squared) of first-differenced hourly time series was computed for each month as an index of shorter-term variability in near-bottom temperatures. Higher short-term variability in temperature should indicate a stronger influence of advective processes in changing both temperature and dissolved oxygen conditions in bottom waters.

### Biological sampling

Thirty adults - of combined male and female specimens - of both the tunicate *Pyura chilensis* (Piure) and the porcelain crab *Allopetrolisthes punctatus* (Changai) were collected from two different sampling locations through scuba diving at about 10 m depth in July (winter) of 2024 February 2025 (summer). Tissue samples were extracted from the digestive gland and gills of each specimen, then stored at −80 °C until DNA extraction. Additionally, to evaluate the ambient microbiota, two samples of water from the same location as the organisms was collected and filtered through a 0.22 μm membrane filter (Isopore™). DNA extraction was performed using the PureLink™ Microbiome DNA Purification Kit (Invitrogen), following the manufacturer’s instructions. DNA quality and concentrations were assessed using a Qubit 4 fluorometer (ThermoScientific, USA). DNA of all individuals was diluted to the same concentration, 100 ng/ul, to perform a two-pool for tissue and organisms of 10 individuals to continue with the 16S rRNA amplification. The sampling design for this study adhered to the three Rs (3Rs) guidelines for animal testing.

### Nanopore library synthesis and sequencing

The complete 16S rRNA gene was amplified from two replicates per sample using the universal primers 27F and 1492R. LongAmp Taq DNA polymerase (New England Biolabs, USA) was utilized for amplification at a reaction volume of 15 μL under the following conditions: 95 °C for 1 min, followed by 30 cycles at 95 °C for 20 s, 56 °C for 30 s, and 65 °C for 1 min, concluding with a final extension at 65 °C for 5 min. The efficacy of the PCR was assessed through 1% agarose gel electrophoresis. PCR products were purified with a 1:2 sample-to-bead ratio using Agencourt AMPure XP beads (Beckman Coulter, USA) for Nanopore library synthesis. The samples were placed in a magnetic stand for washing with freshly prepared 70% ethanol. After rinsing in ultrapure sterile water, the product was separated from the beads by placing it in a magnetic stand. The purified amplicon was quantified again using the Qubit 4 fluorometer (ThermoScientific, USA) and utilized as a template for library synthesis with the 16S rDNA Barcoding Kit (SQK-RAB204, Oxford Nanopore Technologies) following the manufacturer’s instructions. Briefly, the purified 16S rDNA amplicon from a pool of ten individuals was mixed with barcodes. Then, a PCR was performed as stated in the previous step, with a final reaction volume of 50 μL, using the LongAmp Taq Polymerase (New England Biolabs). The PCR product was incubated for 5 min at room temperature using a HulaMixer (ThermoScientific, USA) and subsequently cleaned with Agencourt AMPure XP beads. The purified product was eluted in 10 μL of the elution buffer (10 mM Tris-HCl, pH 8.0, with 50 mM NaCl) following the procedure for washing the magnetic beads. The final concentration of the library was measured using a Qubit 4 fluorometer (Thermo Fisher Scientific, USA), and libraries were tested using High Sensitivity ScreenTape D5000 (Agilent, USA) according to the manufacturer’s instructions on a TapeStation Bioanalyzer 2100 (Agilent, USA). Following the methodology of Oxford Nanopore Technologies, the libraries were pooled in multiplex mode and placed into the flowcell MK1 Spot-ON FLO-MIN107-R9. The ZymoBIOMICS Microbial Community DNA Standard (Cat. No. D6305) was included in the runs. Two biological samples of each tissue, organism, and sample point were sequenced. Sequencing efficiency was monitored using the software MinKNOW 2.0 from Oxford Nanopore Technologies.

### Bioinformatic analysis

Fast5 files were base-called using Guppy (version 3.2.2, Oxford Nanopore Technologies, UK), and a filtering step was applied to retain only sequences with a Q-score of ≥7 (quality filter). The sequencing performance of summer and winter samples was analyzed separately using pycoQC (Leger and Leonardi, 2019). The reads demultiplex, primers, and adapter trimming were performed with Porechop (Wick et al., 2017). Taxonomic classification of reads was performed with EMU v.3.4.5 (Curry et al., 2022). The minimum abundance per sample was filtered at 0.1% using the R package *dplyr* (Wickham et al., 2023). Supplementary Table 1 reports all data used for the analysis (count, taxonomy, abundance, and metadata tables). Diversity patterns and ordination analyses were determined using the R packages *phyloseq*, *vegan*, and *ape* (Dixon, 2003). Taxonomic distribution and distance were analyzed with *phyloseq* and *fantaxtic*, while the statistical comparison was performed with the R package *ggpubr*. The taxonomic abundance comparison among bacterial communities present in each evaluated tissue and organism was performed by heat-trees using *metacoder* R package (Foster et al., 2017). The bacterial community composition was displayed by the Krona visualization tool running in Galaxy (Ondov et al., 2011).

Functional MetaCyc gene pathways prediction (Caspi et al., 2018, 2020) of bacterial communities among organisms, between tissues, and between seasons was performed using PICRUSt2 (Douglas et al., 2020). Read abundances were normalized by 16S rRNA gene copy number, and gene abundances were estimated by multiplying the normalized read counts by the predicted gene copy numbers. Differentially abundant metabolic pathways were analyzed with the STAMP software, using Welch’s *t*-test with a *p*-value cutoff of 0.05 (Parks et al., 2014).

Community interactions among bacterial species were inferred using the network approach (R package SpiecEasi; Sparse Inverse Covariance Estimation for Ecological Association Inference) (Kurtz et al., 2015), and the igraph software (Csardi and Nepusz, 2006). The correlation matrix obtained was filtered using an absolute correlation score greater than or equal to 0.5, where each node represents one species and each edge represents the correlations between the species abundances. Finally, relationships among bacterial communities were identified in gill samples, and their putative functional pathways were determined using the R package mixOmics (Rohart et al., 2017).

## Results

### Oxygen and temperature variability in bottom waters

The time series of temperature and dissolved oxygen concentrations revealed a clear and strong seasonal signal in both variables, with substantial differences in the oxygenation regime of bottom waters between winter and summer months, accompanied by a seasonal change in not only the mean values of temperature but also in its short-term (hourly) variability (Figures 1B,C). From December to March (austral summer), DO levels remained below 2 mg/L for over 60% of the time (Figure 1B). Throughout this period, the frequency distributions of DO were skewed toward low and very low values. Contrasting distributions of DO were observed for the May-August period (autumn-winter), and especially in the June-July window (austral winter), with less than 4% of the recorded DO values below 2 mg/L. Wide and symmetrical distributions of DO values were observed in the transition months of September and April-May (Figure 1B). Exposure to severe hypoxia was highest in December-February, when >75% of the DO values recorded was below 2 mg/L (Figure 1B, red line and symbols). Strong seasonal pattern was also observed for temperature (Figure 1C). However, the minimum and maximum values were not observed in the same months as dissolved oxygen (Figure 1C). The lowest temperatures were recorded in November (spring) with 10.8 °C, and the warmest month was May with 12.5 °C (late autumn). The narrowest range of temperature was observed in June-July (2 °C), when the short-term (hourly) variability of temperature was also minimal (Figure 1C, blue line and symbols). The largest amplitudes in recorded temperature values were observed in December and March (>5 °C), whereas the short-term variability of temperature was highest in February-March (ca. 4 times as in July-August).

### Invertebrates’ microbiota community structure under oxic and hypoxic conditions

**FIGURE 2:**
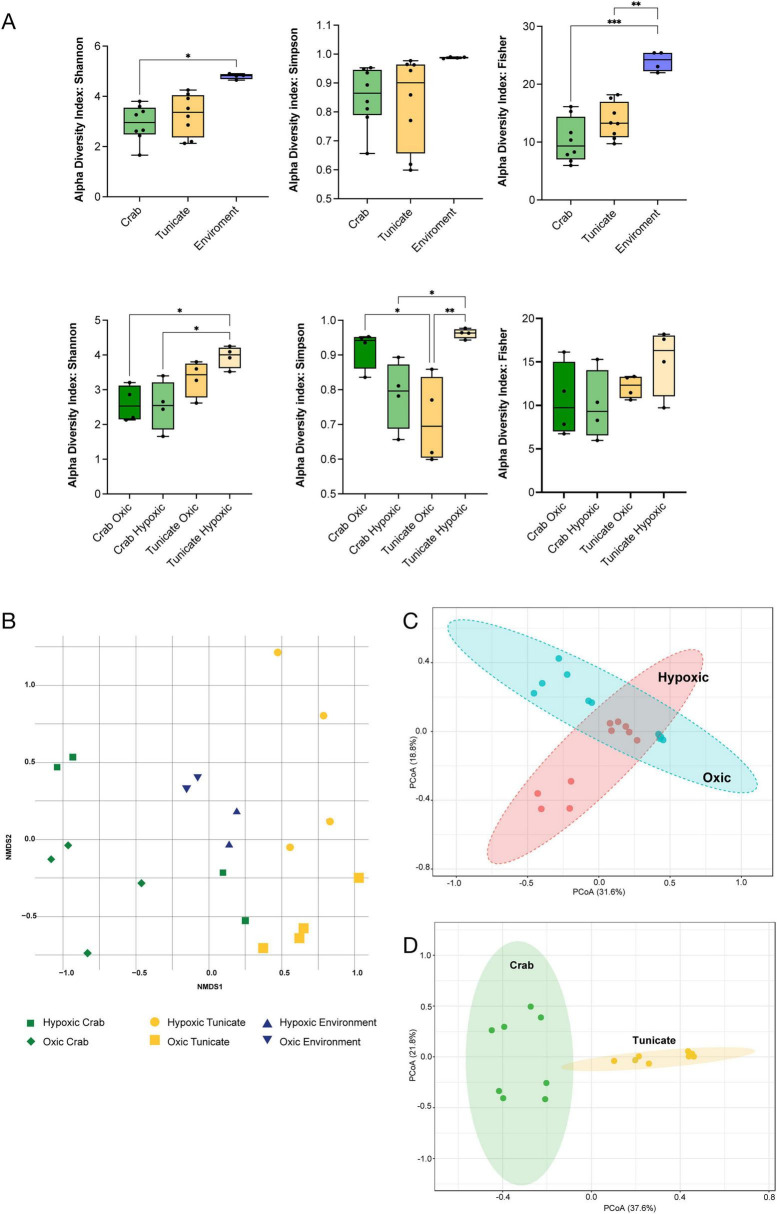
Microbiota composition of Tunicate and Crab tissues obtained from Chome during the summer (hypoxic) and winter (oxic). **(A)** Alpha diversity was assessed using Shannon, Simpson and Fisher diversity indices; **(B)** Nonmetric multidimensional scaling (NMDS) for environmental analysis of Tunicate and Crab microbiota; **(C)** Beta diversity examined through principal coordinates analysis (PCoA) based on Bray-Curtis distances of hypoxic and oxic condition (PERMANOVA *F*-value: 3.15, R^2^: 0.14, *p*-value: 0.012); **(D)** Beta diversity was examined through principal coordinates analysis (PCoA) based on Bray-Curtis distances of Tunicate and Crab (PERMANOVA *F*-value: 7.37, R^2^: 0.34, *p*-value: 0.001). *p-value <0.05, **p-value <0.01, ***p-value <0.001.

A total of 2,659,031 reads were obtained after trimming and filtering, comprising 1,254,397 for the summertime, hypoxia-dominated conditions and 1,404,634 reads for the wintertime oxygenated conditions (Supplementary Figure 1 and Supplementary Tables 2, 3). Using the data of both tissues per species alpha diversity analyses revealed significant differences among the animals’ microbiota and the environment, where is observed more diversity with significant differences according to the Shannon and Fisher index (Figure 2A). Relate with the oxygenation condition, significant differences in Shannon index were observed between the crab and the tunicate under hypoxic conditions (Figure 2A). Simpson index also demonstrates significant differences between the crab and the tunicate under oxic and hypoxic condition. Also, the microbiota diversity in the tunicate is highly differences when is compare oxic and hypoxic condition according Simpson index (Figure 2A). A Nonmetric Multidimensional Scaling (NMSD) analysis indicated differences in the community structure of bacteria in the microbiota and those in the ambient microbial communities (ANOSIM *R* = 0.9, *p* < 0.002, Figure 2B), consistent with differences between species (Figure 2B). Similarly, there were differences in the bacterial community structure across seasons (Principal Coordinates Analysis; PCoA, Figure 2C) and organism (Figure 2D). However, there were no apparent shifts in the microbiota of tunicates and crabs when comparing between oxygenation regimes (Supplementary Figure 2).

### Compositional changes in the bacterial community during oxic and hypoxic conditions

**FIGURE 3:**
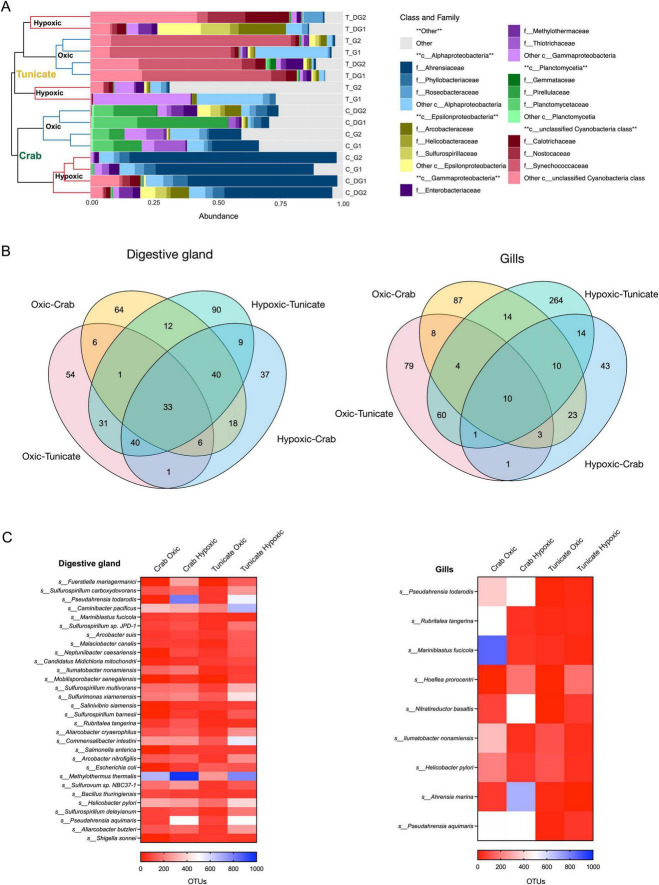
Families and species of bacteria identified from the digestive gland and gill tissues of the Tunicate and Crab. **(A)** The top five taxa by class and family are shown. Tunicate samples are indicated in orange, while the crustacea Crab samples are highlighted in green. (H) hypoxic, (O) oxic, DG: digestive gland, (G) gills; **(B)** Venn diagram representation of the number of bacteria species identified in the digestive gland and gill tissues of both organisms under oxic and hypoxic conditions; **(C)** OTUs for common bacteria species represented in a heatmap. **p-value <0.01.

Differences in microbiota communities’ abundance were observed in the digestive gland and gill tissues between the tunicate and the crab (Figure 3). In the tunicate *P. chilensis*, the bacterial community at family level exhibited three clades: two for hypoxia, one each for gills and digestive gland bacteria, and another clade associated with oxic conditions that also separates both tissues (Figure 3A). Under both oxygenation regimes, the most abundant bacterial family in the tunicate’s microbiota is Nostocaceae from the Cyanobacteria order (Figure 3A). As for the crab *A. punctatus*, its bacterial community at family level separated into two main clades: hypoxic and oxic, also exhibiting differentiation between digestive gland and gill tissues (Figure 3A). The family Ahrensiaceae becomes prominent under hypoxic conditions while the family Gemmateceae dominates under oxic regime (Figure 3A). Notably, bacteria from the families Methylothermaceae and Thiotrichaceae were identified in both species under both oxygenation conditions.

A total of 33 bacterial species constitute the digestive gland core, while 10 bacterial species were identified for the gill core (Figure 3B). The tunicate’s digestive gland revealed 90 and 54 exclusive species during the hypoxic and oxic periods, respectively. Samples from the crab’s digestive gland 37 and 64 exclusive species in hypoxic and oxic conditions, respectively (Figure 3B). In the gills, the tunicate exhibited the highest number of exclusive bacterial species during the hypoxia-dominated period (264 species, Supplementary Table 4). In contrast, during the oxygenated season, there were only 79 exclusive bacterial species in the tunicate’s microbiota (Figure 3B). In the crab’s gills, 87 and 43 exclusive bacteria were identified under oxic and hypoxic conditions, respectively (Figure 3B). A core of bacteria was identified among species and oxygen regimes for each tissue with 33 bacterial species in the digestive gland core, and 10 in the gills core, with abundance differences among the oxygen condition and organism (Figure 3C).

#### Shifts in bacterial community interactions during oxic and hypoxic conditions

**FIGURE 4:**
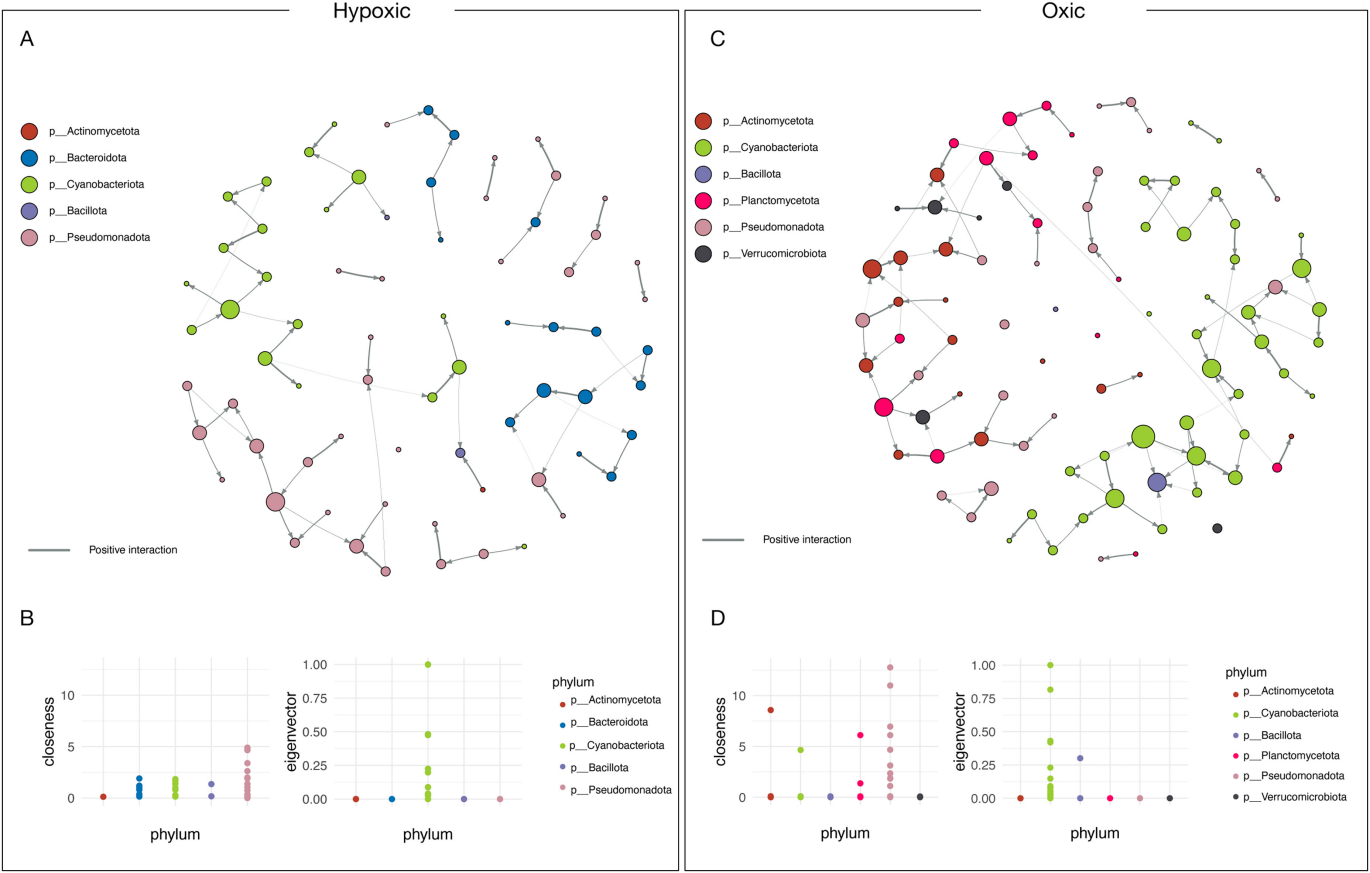
Network analysis of bacteria species interactions during hypoxic and oxic **(A–C)**. Node centrality analysis of the key taxa phylum **(B–D)**. FDR-adjusted *p*-value cutoff of 0.05 and a log LDA score of –4.

Network analyses were conducted to unveil bacterial co-occurrence. The network displayed greater complexity when animals were exposed to oxic compared to hypoxic conditions (Figure 4). Under hypoxia-dominated conditions, there were five hubs (i.e., key taxa by phylum) involving interaction among Actinomycetota, Bacteroidota, Cyanobacteriota, Bacillota, Pseudomonadota (Figure 4A). The taxa with the highest interaction (i.e., closeness) were Bacteroidota and Cyanobacteriota (Figure 4B). Under oxygenated conditions, six hubs were observed: the phyla Actinomycetota, Cyanobacteriota, Bacillota, Planctomycetota, Pseudomonadota, and Verrucomicrobiota (Figure 4C). Interestingly, Cyanobacteriota exhibited the highest node influence in the connected network (i.e., eigenvector) for both oxygenation regimes (Figures 4B,D). At the species level, *Salmonella enterica*, *Escherichia coli*, and *Pseudahrensia todarodis* exhibited the highest closeness levels under hypoxia. Meanwhile, *Pseudomonata* species such as *Pluralibacter pyrinus* and *Helicobacter pylori* displayed high values in oxic conditions.

Using the date of both organisms, oxygen condition-specific microbiota was identified in gills and digestive glands (Supplementary Figure 3 and Supplementary Table 5). Under hypoxic conditions, 56 species were annotated, representing bacterial classes such as Alphaproteobacteria, Bacteroidia, and Flavobacteriia (Supplementary Figure 3 and Supplementary Table 6). Under oxygenated conditions, 58 bacterial species were present in both tissues; the most abundant bacterial class was Cyanobacteria, primarily represented by the genus *Synechococcus*, which dominated under this regime (Supplementary Figure 3 and Supplementary Table 5).

### Functional prediction of the host microbiota

**FIGURE 5:**
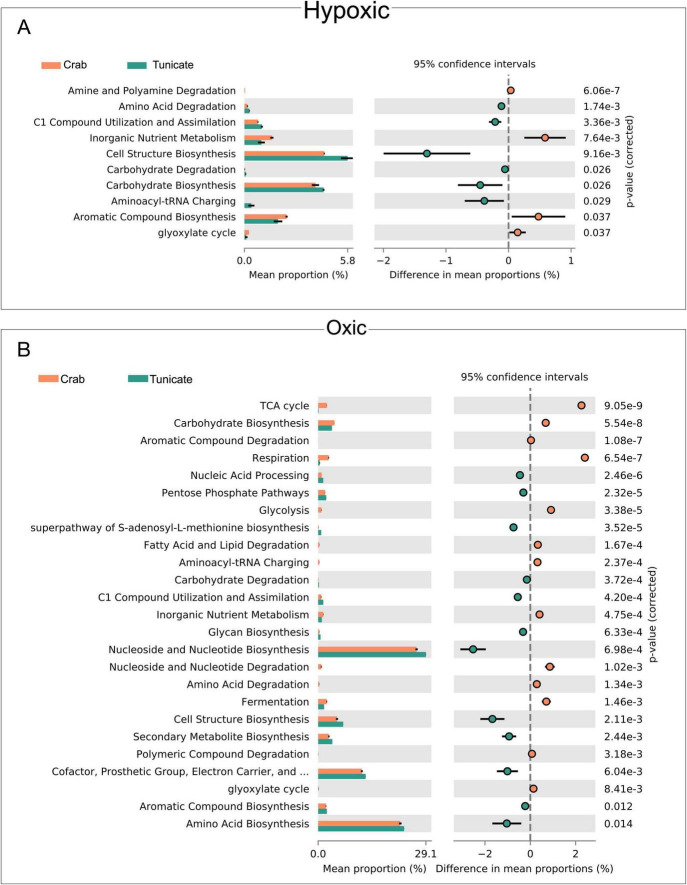
Functional prediction of the microbiota for hypoxic and oxic conditions inferred using PICRUSt2. The mean proportion of top MetaCyc gene pathways of the Crab and Tunicate microbiota analyzed with statistical inference using STAMP software during **(A)** hypoxic and **(B)** oxic conditions.

Using the data of both tissues per species, a functional prediction of MetaCyc gene pathways was conducted from the identified bacterial species (Nanopore data using Emu; Supplementary Table 6). In terms of putative microbiota function in hypoxic conditions, ten gene pathways were significantly different between the tunicate and the crab under hypoxic conditions, including amino acid degradation, cell structure biosynthesis, and carbohydrate biosynthesis/degradation (Figure 5A). Under oxic conditions 20 pathways were identified with significant differences between the tunicate and the crab (Figure 5B). Among the highly represented gene pathways are Nucleoside and Nucleotide Biosynthesis, Amino Acid Biosynthesis, and Cofactor-Prosthetics Group-electron Carrier Cofactor-and Vitamin Biosynthesis (Figure 5B).

**FIGURE 6:**
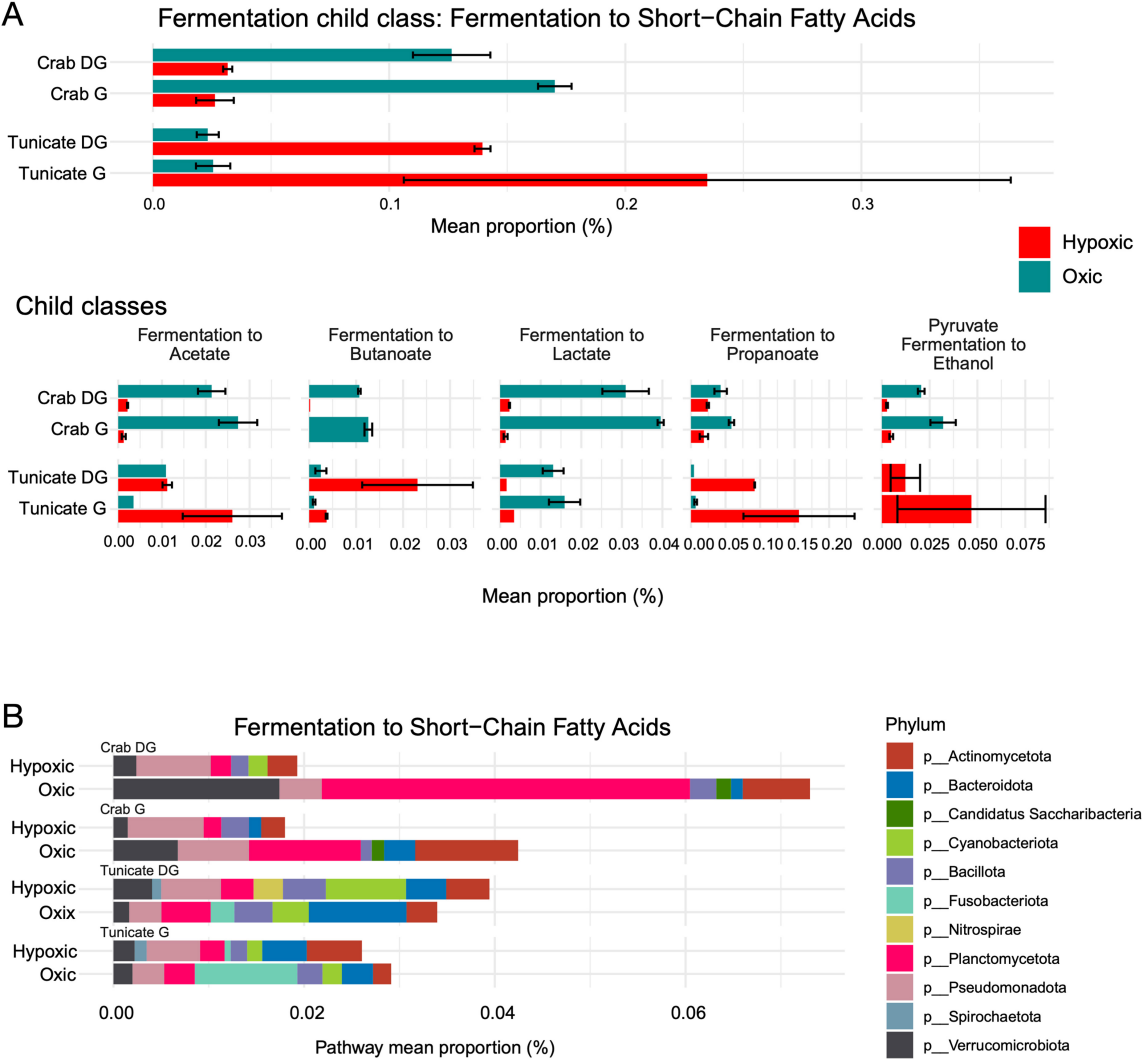
Functional prediction of microbiota inferred using PICRUSt2. **(A)** The MetaCyc gene pathway for fermentation to short-chain fatty acids abundance is represented, along with the subpathway for fermentation to butanoate abundance. **(B)** The phylum associated with the MetaCyc gene pathway for short-chain fatty acids abundance is also included.

Notably, the fermentation of short-chain fatty acids appeared in a higher proportion in the tunicate’s tissues during hypoxia, compared to the crab under the same conditions (Figure 6A). Furthermore, the tunicate exhibited a greater potential for the fermentation of acetate, butanoate, propanoate, and pyruvate to ethanol during hypoxia, whereas the crab displayed a higher potential for these metabolic processes under oxic conditions (Figure 6A). A high proportion of bacteria associated fermentation of short-chain fatty acids were observed in both organism under hypoxia and oxic conditions, for instance, Planctomycetota, Verrucomicrobiota, Actinomycetota, and Pseudomonadota which are involved in butanoate fermentation in the gill tissues of both species (Figure 6B).

Finally, a network analysis was conducted to explore the relationships between the most abundant gill microbiota families and the MetaCyc gene pathways. For the crab’s microbiota, Vibriocaceae found under hypoxic conditions were positively correlated only with Glycolysis and negatively correlated with most of the MetaCyc gene pathways, including the TCA cycle, fermentation, carbohydrate biosynthesis, and inorganic nutrient metabolism (Supplementary Figure 4). In contrast, the Enterobacteriaceae, Roseobacteraceae, Helicobacteraceae, and Bartonellaceae families were positively correlated with most of the MetaCyc gene pathways (Supplementary Figure 4). For the microbiota of tunicate gills, the bacterial families observed under hypoxic and oxic conditions were positively correlated with MetaCyc gene pathways (Supplementary Figure 4). For instance, the bacteria of the Roseobacteraceae family under hypoxic conditions are positively correlated with amino acid degradation. In the case of microbiota under oxic conditions, the Clostridiaceae, Carnobacteriaceae, Corynebacteriaceae, and Xanthomonadaceae families exhibited a positive correlation with Carbohydrate Degradation, Secondary Metabolite Biosynthesis, Cofactor Prosthetic Groups, Electron Carriers, and Vitamin Biosynthesis.

## Discussion

Microbial communities play a central role in marine biogeochemical cycles. Their composition responds to environmental variability over various spatial and temporal scales. Although efforts have been made to understand spatial/biogeographical patterns (Zinger et al., 2011), successional processes (Teeling et al., 2012), and responses to hypoxia in environmental microbial communities (Jessen et al., 2017), little is known about the responses of microbiota in marine organisms to changes in critical environmental variables. The ubiquity of microorganisms in the environments in which animals are immersed suggests a functional connection between the ambient microbial community and the well-being of various hosts. For instance, juvenile invertebrates or fish larvae deprived of their gut microbiota experience adverse effects on their growth and survival (Rawls et al., 2004; Sison-Mangus et al., 2015). The role of microbial communities in marine ecosystem functioning is unequivocal, with microorganisms being key drivers of the ocean’s biogeochemistry and having a critical role in maintaining animal health (Banerjee et al., 2018).

Dissolved oxygen is a primary driver of coastal animal benthic community structure, behavior, physiology, and ultimately species survival (Bremner et al., 2006). In recent years, ocean deoxygenation has increased, undermining the adaptation and survival of marine organisms worldwide (Cárcamo et al., 2017; Hernández and Tapia, 2021; Hernández-Miranda et al., 2012). The Chilean coast experiences seasonal upwelling, leading to recurring environmental hypoxia, especially during the summer (Hernández-Miranda et al., 2012; Muñoz et al., 2025). Our study area was chosen as a representative site for the seasonal and shorter-term variation in dissolved oxygen levels that are forced mainly by wind-driven upwelling, but also by the interaction of coastal flows - including tidal currents - and the local topography (Sobarzo et al., 2016). Thus, conditions at our study site ranged between moderate to severe hypoxia during the austral spring-summer season and well-oxygenated conditions in the austral winter. Here, variations in the microbiota of model benthic organisms, such as tunicates and decapod crustaceans, were evaluated to elucidate their role in marine organism adaptation to environmental hypoxia. Upwelling-driven hypoxia is coupled with shifts in primary productivity and turbidity. For filter-feeding tunicates like *P. chilensis*, the distribution of primary producers likely drives Cyanobacteria dominance during high light transmittance. Conversely, the influx of Ahrensiaceae in crabs during hypoxia could be explained to sediment resuspension. Thus, while feeding strategies and particle distribution play a role in the microbiota, dissolved oxygen remains the primary physiological driver.

Microbiota studies in ascidians (da Silva Oliveira et al., 2013) and crabs (Bacci et al., 2023) have reported species-specific microbiota composition. Also, microbiota variation has been reported in different marine organisms in response to low-oxygen levels (Khan et al., 2018; Montúfar-Romero et al., 2025; Ullah Khan et al., 2021). Here, the microbiota diversity in the Tunicate is more affected by oxygen conditions than in the Crab. Interestingly, in the Tunicate, microbiota diversity increases under hypoxic conditions, which suggests a role for this microbiota in the Tunicate’s adaptation to low oxygen availability. In the Chinese mitten crab (*Eriocheir sinensis*), changes in intestinal microbiota have been reported in response to hypoxia (Deng et al., 2024), with a high dominance of the Bacillota and Bacteroidota phyla. At the class level, the authors reported a decrease in Gammaproteobacteria in the hypoxia-exposed group compared with the control (Deng et al., 2024). In our study, the class Gammaproteobacteria was more abundant in samples under hypoxic conditions than under oxic conditions. In another study, performed in the oyster *Crassostrea hongkongensis*, a significant increase in Pseudomonadota abundance was reported under low-oxygen conditions (Xie et al., 2023). Interestingly, the microbiota in the tunicate’s gill displayed a high abundance of Bacteroidota bacteria under hypoxic conditions, compared to what has been reported in the Chinese mitten crab. Moreover, Bacilli bacteria have been associated with hypoxia in Chinese mitten crab (Deng et al., 2024), and oysters (Xie et al., 2023), suggesting that those bacterial groups are common among marine invertebrates exposed to hypoxia. At the tissue level, it is important to note that, while the gill tissue is permanently exposed to ambient waters and, therefore, the associated changes in oxygenation levels, the digestive gland is contained and, therefore, under permanent hypoxic to anoxic conditions. Consistently, in our study, Bacillota were observed in the tunicate’s digestive gland in both seasons (hypoxia-dominated and oxic conditions), similar to what was reported in the mussels *Mytilus couscous* (*M. couscous*) (Ullah Khan et al., 2021) and *M. chilensis* (Montúfar-Romero et al., 2025).

Microbiota interactions play a crucial role in maintaining ecological balance, and a robust microbial network (i.e., high connectivity and sub-nodes) is associated with a balanced microbiota with a positive impact on the host (Codello et al., 2022). For example, a study on the fish *Bostrichthys sinensis* showed less robust microbiota networks under hypoxic conditions than those exposed to oxic conditions (Fan et al., 2020). Overall, our model species exhibited more nodes and sub-nodes in oxic than in hypoxic conditions, suggesting that hypoxic stress affects both the host and the microbiota.

Hypoxic stress triggers a cascade of metabolic effects leading to a high energy demand, which can induce changes in the organism’s response to stressors, including pathogens or even affect digestion regulation (Chen et al., 2022; Xie et al., 2023). Due to the microbiota’s control over several host processes at the metabolic level (Bäckhed, 2011; Lindsay et al., 2020; Tremaroli and Bäckhed, 2012), understanding its function is essential for grasping the regulation of host metabolism. Even though assessing gene expression is beyond the scope of this study, the functional prediction analysis of the microbiota revealed the potential for various metabolic functions. In this context, environmental hypoxia appeared to promote processes such as carbohydrate biosynthesis, degradation, and amino acid degradation. This suggests that reduced oxygen levels have an impact beyond the known physiological stress of marine animals subjected to hypoxia, but also at the microbiota level in terms of composition and functioning. This aligns with observations conducted on the benthic fish *Bostrichthys sinensis*, in which similar functional network differences were reported comparing control (oxic) to hypoxic conditions (Fan et al., 2020).

In humans, the short-chain fatty acids (SCFAs) produced by gut microbiota play multiple roles (Topping and Clifton, 2001). Despite the taxonomic dominance of Cyanobacteria in the tunicate under oxic conditions, our functional predictions - normalized for gene copy number - distinctly identified an enrichment of SCFA fermentation pathways during hypoxia. This suggests that the predicted metabolic shift toward anaerobic functions (i.e., butanoate and acetate production), is a response to hypoxia independently of the dominant aerobic taxa. Moreover, SCFAs have been associated with stabilizing the hypoxia-inducible factor (HIF) gene in epithelia with healthy microbiota (Wang R. X. et al., 2021). This occurs under limited oxygen concentrations, triggering processes such as energy metabolism, inflammation, and apoptosis (Greijer and Van der Wall, 2004; Lee et al., 2020). Although this mechanism has not been reported for invertebrates, high abundances of bacteria associated with butyrate fermentation were found under hypoxia within the tunicate’s digestive gland, suggesting a similar regulatory role of HIF in invertebrates. This type of response would probably prevent cell apoptosis and aid the host organism’s adaptation to prolonged hypoxia. This aligns with the role of butyrate as a histone deacetylase inhibitor and regulator of intestinal genes (Davie, 2003; Wang et al., 2020). Indeed, butyrate inhibits the PHDs (pyruvate dehydrogenase) gene in human microbiota and stabilizes HIF (Wang R. X. et al., 2021). Altogether, this suggests a crucial symbiotic relationship where the microbiota’s metabolic byproducts, such as butyrate, are key to the host’s physiological adaptation to low-oxygen environments.

Although a stable core of species persists across seasons reaching 33 in the digestive gland, 10 in the gills, the microbiota is primarily characterized by large-scale shifts in overall community structure and the recruitment of exclusive species during hypoxia. Thus, the non-core community reacts to a changing environment. Upwelling-driven environmental variability in coastal waters, particularly the seasonal hypoxia that characterizes the shores of central Chile and other Eastern Boundary regions, profoundly impacts animal physiology at both the host and microbiota levels. These impacts lead to a drastic reduction in microbial diversity and significant shifts in community composition, favoring anaerobic and facultative anaerobic taxa in the exposed gills and, interestingly, also in the permanently hypoxic micro-environment of the digestive glands. Crucially, oxic conditions host a different microbial community and function, indicating persistent alterations rather than transient perturbations. These microbial shifts are likely to alter metabolic functions, especially within the host gut, with considerable implications for the host’s health. While microbiota compositional shifts are clearly identified, potential functions are predicted with their limitations (Sun et al., 2020). Thus, future studies are required, for instance, to use a common-garden design to evaluate, under controlled conditions, changes in the microbiome, and to include metatranscriptome and metabolome analyses to determine whether the functional consequences inferred in this study are consistent. Finally, understanding the effects of cumulative stress, particularly the response to recurrent environmental factors such as hypoxia, is vital for accurately predicting animal adaptation and the long-term resilience and health of aquatic ecosystems. Here, model invertebrate species from subtidal waters along the Chilean coast, and their microbiotas, can shed light on how coastal organisms are coping with the local manifestations of climate change and anthropogenic pollution.

## Data Availability

The datasets presented in this study can be found in online repositories. The names of the repository/repositories and accession number(s) can be found in the article/Supplementary material.

## Supplementary material

Supplementary Figure 1 Rarefaction curve of raw data was obtained for each sample.

Supplementary Figure 2 Microbiota diversity for the Crab and Tunicate gills and digestive gland was determined by Alpha diversity, which was assessed using Shannon, Simpson, and Fisher diversity index.

Supplementary Figure 3 Microbiota presented in gills and digestive gland tissues during hypoxic and oxic conditions. **(A)** The upset plot showed the number of bacterial species present and shared in both tissues. **(B)** Bacteria were identified at the genus level in gills and the digestive gland under each oxygen condition.

Supplementary Figure 4 Network analysis of bacterial families and the Top MetaCyc gene pathways designated for the gill microbiota of both analyzed organisms. Red: bacteria families in a hypoxic condition; Green: bacteria families in an oxic condition. The red lines indicate negative correlations, and the green lines indicate positive correlations.
