# Supplementary material for: Microbiota responses to environmental stress in mobile and sessile marine invertebrates: evidence for the effect of dissolved oxygen variations
Source: Front Microbiol. 2026 May 13;17:1764313. doi: 10.3389/fmicb.2026.1764313 (PMC13215113; doi:10.3389/fmicb.2026.1764313)
Supplement: Supplementary file 1 [file Supplementary_file_1.zip › Table S2 and Table S3.docx]

**Supplementary Tables.**

**Table S2:** Reads distribution per barcode follow the demultiplex and trimming data using Porechop.

| Hypoxic/summer | | | |  | Oxic/winter | | | |
| --- | --- | --- | --- | --- | --- | --- | --- | --- |
| Barcode | Organism | Tissue | Poreshop Reads | | Barcode | Organism | Tissue | Poreshop Reads |
| BC15 | Mock | - | 25.385 |  | BC11 | Mock | - | 65.365 |
| BC09 | Piure | Gills | 44.498 |  | BC01 | Piure | Gills | 30.651 |
| BC21 | Piure | Gills | 52.856 |  | BC02 | Piure | Gills | 132.610 |
| BC22 | Piure | Digestive Gland | 160.281 |  | BC03 | Piure | Digestive Gland | 125.381 |
| BC23 | Piure | Digestive Gland | 50.684 |  | BC04 | Piure | Digestive Gland | 59.633 |
| BC05 | Changai | Gills | 120.831 |  | BC05 | Changai | Gills | 150.644 |
| BC06 | Changai | Gills | 80.060 |  | BC06 | Changai | Gills | 52.521 |
| BC14 | Changai | Digestive Gland | 52.821 |  | BC07 | Changai | Digestive Gland | 170.074 |
| BC24 | Changai | Digestive Gland | 244.621 |  | BC08 | Changai | Digestive Gland | 381.533 |
| BC17 | Water | Water | 92.559 |  | BC09 | Water | Water | 89.766 |
| BC20 | Water | Water | 329.801 |  | BC10 | Water | Water | 145.456 |
| Total |  |  | 1.254.397 |  | Total |  |  | 1.403.634 |

**Table S3**. Taxonomic assignment per barcode of samples collected during summer and winter seasons.

| Barcode | Organism | Tissue | Oxygen condition | Assignment (reads) | Assignment (%) | Total tax_id* |
| --- | --- | --- | --- | --- | --- | --- |
| BC15 | Mock |  |  | 635 | 2.5 | 13 |
| BC09 | Piure | Gills | Hypoxia | 36,027 | 80.96 | 202 |
| BC21 | Piure | Gills | Hypoxia | 26,513 | 50.16 | 295 |
| BC22 | Piure | Digestive Gland | Hypoxia | 21,579 | 13.46 | 174 |
| BC23 | Piure | Digestive Gland | Hypoxia | 30,216 | 59.62 | 151 |
| BC05 | Changai | Gills | Hypoxia | 47,185 | 39.05 | 99 |
| BC06 | Changai | Gills | Hypoxia | 46,728 | 58.37 | 60 |
| BC14 | Changai | Digestive Gland | Hypoxia | 40,419 | 76.52 | 100 |
| BC24 | Changai | Digestive Gland | Hypoxia | 41,485 | 16.96 | 142 |
| BC17 | Water | Water | Hypoxia | 50,910 | 55 | 219 |
| BC20 | Water | Water | Hypoxia | 57,829 | 17.53 | 227 |
| Total |  |  |  | 399,526 | 31.85 | 675 |
| BC11 | Mock |  |  | 1,606 | 85.08 | 21 |
| BC01 | Piure | Gills | Oxic | 16,929 | 55.23 | 126 |
| BC02 | Piure | Gills | Oxic | 76,179 | 57.45 | 108 |
| BC03 | Piure | Digestive Gland | Oxic | 34,258 | 27.32 | 156 |
| BC04 | Piure | Digestive Gland | Oxic | 9,052 | 15.18 | 105 |
| BC05 | Changai | Gills | Oxic | 121,275 | 80.5 | 350 |
| BC06 | Changai | Gills | Oxic | 45,362 | 86.37 | 141 |
| BC07 | Changai | Digestive Gland | Oxic | 61,868 | 36.38 | 84 |
| BC08 | Changai | Digestive Gland | Oxic | 35,487 | 9.3 | 166 |
| BC09 | Water | Water | Oxic | 49,958 | 55.65 | 273 |
| BC10 | Water | Water | Oxic | 71,473 | 49.14 | 235 |
| Total |  |  |  | 577,451 | 41.14 | 567 |

*number of tax_id after the filter of minimum abundance of 0,1%.
