# Supplementary figures and images for: Microbiota responses to environmental stress in mobile and sessile marine invertebrates: evidence for the effect of dissolved oxygen variations
Source: Front Microbiol. 2026 May 13;17:1764313. doi: 10.3389/fmicb.2026.1764313 (PMC13215113; doi:10.3389/fmicb.2026.1764313)

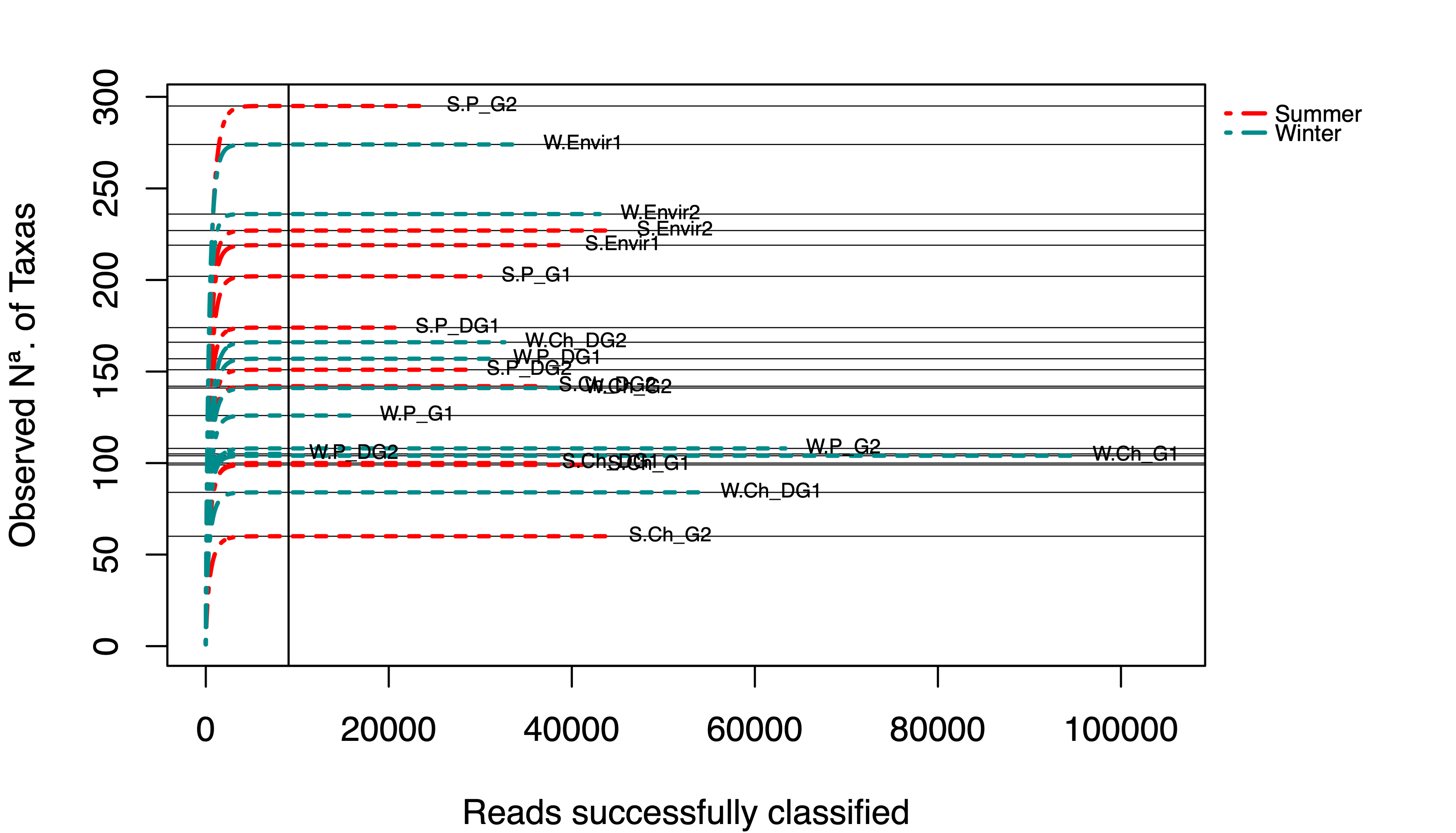

Supplement: Supplementary file 1 [file Supplementary_file_1.zip › Supplementary Figure 1.tiff]

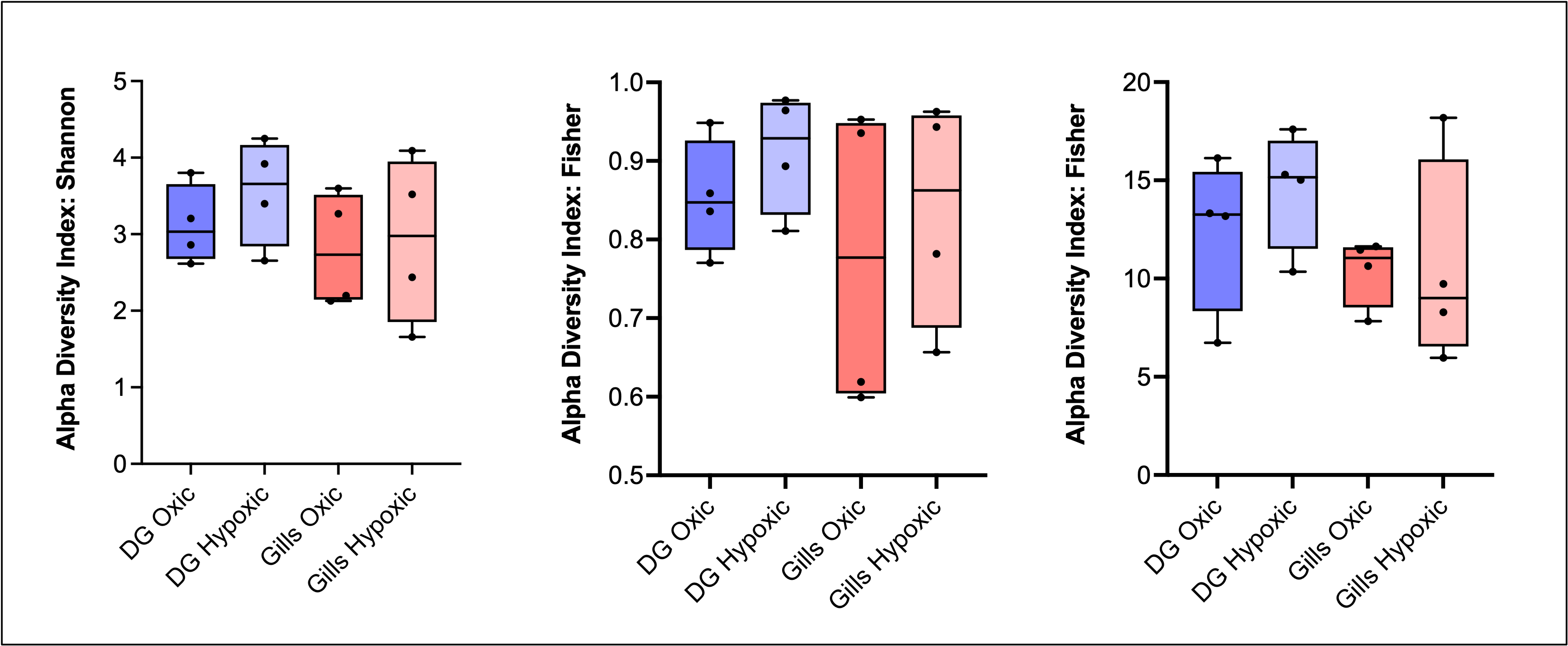

Supplement: Supplementary file 1 [file Supplementary_file_1.zip › Supplementary Figure 2.tiff]

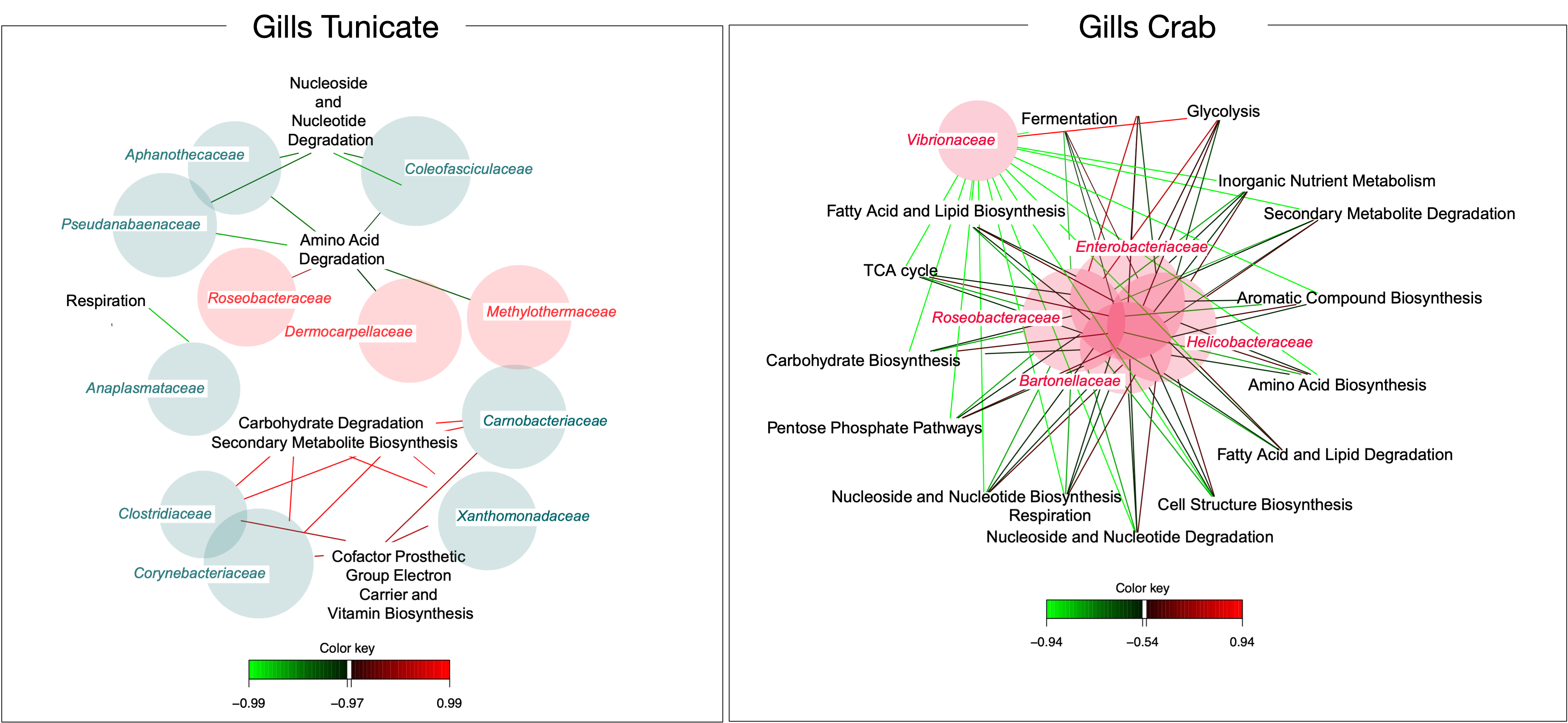

Supplement: Supplementary file 1 [file Supplementary_file_1.zip › Supplementary Figure 4.tiff]
